# Sarcoïdose systémique: cause exceptionnelle de calcifications surrénaliennes

**DOI:** 10.11604/pamj.2014.18.170.4769

**Published:** 2014-06-20

**Authors:** Wafa Chebbi, Saida Jerbi

**Affiliations:** 1 Service de Médecine Interne, CHU Taher Sfar Mahdia, 5100 Mahdia, Tunisie; 2 Service de Radiologie, CHU Taher Sfar Mahdia, 5100 Mahdia, Tunisie

**Keywords:** Sarcoïdose, calcifications surrénaliennes, corticothérapie, Sarcoidosis, adrenal calcification, corticotherapy

## Image in medicine

La sarcoïdose est une granulomatose systémique dont les localisations préférentielles sont les poumons, le système lymphatique et la peau. Cependant, l'atteinte des glandes endocrines est rarissime et la localisation surrénalienne est exceptionnelle. Nous rapportons l'observation d'une patiente âgée de 39 ans, sans antécédents pathologiques, adressée pour bilan étiologique d'une uvéite granulomateuse de l'oe'il gauche. A l'anamnèse, la patiente rapportait une toux sèche associée à une dyspnée d'effort, évoluant depuis 3 mois. Il n'y avait pas de notion de contage tuberculeux, ni de fièvre, ni d'altération de l’état général et ni de traumatisme abdominal. L'examen clinique était normal. Le bilan biologique montrait une hypercalcémie à 2,8 mmo/l, une hypercalciurie à 0,2 mmol /kg /24 heures et une élévation de l'enzyme de conversion de l'angiotensine à 104 UI/L (normale: 12- 68). La recherche de mycobactéries par tubage gastrique et dans les urines était négative. L'intradermo-réaction retrouvait une anergie tuberculinique et le quantiféron était négatif. Le scanner thoraco-abdominal objectivait une infiltration parenchymateuse pulmonaire faite de micronodules et de nodules pleuraux et péri-lobulaires bilatéraux prédominant dans les régions moyennes et supérieures sans atteinte fibrosante avec des adénopathies hilaires, latéro-trachéales bilatérales et sous carinaires non calcifiées, non nécrotiques et non compressives. A l’étage abdominal, il y avait des calcifications surrénaliennes gauches compatibles avec des séquelles d'atteinte granulomateuse. La biopsie de glandes salivaires montrait des lésions épithélioïdes et giganto-cellulaires sans nécrose caséeuse. La cortisolémie de base était normale (166 µg/dl). Le diagnostic de sarcoïdose systémique était retenu. Une corticothérapie à la dose de 0,5 mg/kg/j était instaurée avec une évolution favorable.

**Figure 1:**
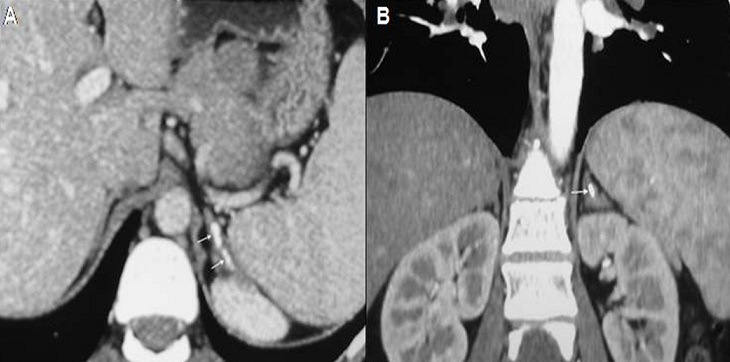
TDM abdominale. (A) dans le plan axial et (B) en reconstruction coronale : calcifications surrénaliennes gauches (flèches)

